# Cognitive and Spontaneous Brain Activity in Nonaddictive Smartphone Users Among Older Adults in China: Cross-Sectional Study

**DOI:** 10.2196/63485

**Published:** 2025-07-24

**Authors:** Zhenyu Wan, Xucong Qin, Qirong Wan, Baohua Xu, Hong Lin, Fangcheng Ouyang, Gaohua Wang

**Affiliations:** 1 Department of Psychiatry, Renmin Hospital of Wuhan University, No. 99 Zhangzhidong Street, Wuchang District, Wuhan, AH 553, China, 86 15872722863; 2 Department of Psychiatry, Yichang City Clinical Research Center for Mental Disorders, Yichang, China; 3 Department of Psychiatry, BoDe Psychiatric Hospital, Yichang, China

**Keywords:** older adults, aged, cognition, mental health, smartphone, fMRI, functional magnetic resonance imaging, mobile phone

## Abstract

**Background:**

The effects of smartphone use on mental health and brain activity in adolescents have received much attention; however, the effects on older adults have received little attention. As more and more older adults begin to use smartphones, exploring the effects of nonaddictive smartphone use on mental health, cognitive function, and brain activity in older adults is imperative.

**Objective:**

This study aimed to examine differences in cognitive performance, emotional symptoms (depression, anxiety, and insomnia), and brain functional activity between older adults who use smartphones and those who do not.

**Methods:**

A total of 1014 community-dwelling older adults aged 60 years and above were surveyed in a rural area of China. Participants were categorized into 2 groups based on their smartphone use status. The Patient Health Questionnaire, Generalized Anxiety Disorder Scale, Insomnia Severity Index, and Montreal Cognitive Assessment-Basic were used to evaluate the symptoms of depression, anxiety, insomnia, and cognitive function of the participants by trained medical staff. To explore neural mechanisms, a subsample of 130 participants (89 smartphone users and 41 nonusers) was selected using stratified random sampling for resting-state functional magnetic resonance imaging scanning. Participants with contraindications for magnetic resonance imaging (eg, metal implants or claustrophobia) or who refused to participate were excluded. Functional brain activity was analyzed and compared between groups.

**Results:**

Among all 1015 older adults, 641 reported using smartphones, while 373 reported never using smartphones. Older adults who use smartphones exhibited better cognitive function compared with those who never use smartphones (*z*=3.806, *P*<.001), especially in the domains of fluency (*z*=3.025, *P*=.002) and abstraction (*z*=5.311, *P*<.001). However, there were no significant differences in levels of depression (*z*=0.689, *P*=.49), anxiety (*z*=0.934, *P*=.35), and insomnia (*z*=0.340, *P*=.73). In terms of the magnetic resonance imaging findings, a total of 130 participants completed functional magnetic resonance imaging scanning, including 89 who use smartphones and 41 who never use smartphones, and results showed that older adults who were smartphone users exhibited higher degree centrality values in the left parahippocampal gyrus.

**Conclusions:**

These findings suggest that smartphone use among older adults is associated with better cognitive performance and fewer emotional symptoms, potentially linked to enhanced brain activity in key cognitive regions. Promoting digital engagement may offer cognitive and emotional benefits for aging populations. Longitudinal studies are warranted to examine causal relationships.

## Introduction

The aging of the population has become a global issue. According to the World Population Prospects 2022, published by the United Nations Population Division, the older population is projected to reach 994 million by 2030 worldwide [1]. Aging means a decline in physical ability and function, and in the process, they gradually become disconnected from society [2], participate less in social activities [3], and sleep duration and quality decline significantly [3,4]. At the same time, the aging process is associated with a high risk of cognitive impairment [5], encompassing various cognitive domains such as reaction time, sensory processing, attention, memory, reasoning, and executive functioning [6]. This cognitive aging not only affects their daily life abilities but also has the potential to lead to dementia ultimately [7]. Additionally, a decline in physiological functioning and a decrease in social participation are associated with a higher risk of depression in older adults [8], which impacts the quality of life of affected individuals and their families and presents a considerable challenge for society as a whole. Therefore, there is increasing attention given to how to improve cognitive function and reduce the risk of depression among older adults.

As an important invention of the 21st century, smartphones have become an indispensable part of people’s lives. In recent years, the application scenarios and user demographics of smartphones have expanded, with many older adult individuals in rural areas also beginning to use smartphones for simple recreational activities and socializing through chat applications [9]. While researchers have long focused on the effects of smartphone use on mental health and cognitive function, research has focused on adolescents, and the effects on older adults have been less explored. Current studies of adolescents have generally linked smartphone use to reduced sleep duration, decreased sleep quality, and a higher risk of depression [10,11]. However, it should be emphasized that this is largely due to the addictive use of smartphones [12,13]. Objectively speaking, as an important tool in daily life and work, mobile phones play an irreplaceable role. Healthy use of mobile phones can help individuals broaden their horizons, seek support for emotional needs, provide opportunities for leisure and entertainment, and may improve their cognitive functioning. Compared with adolescents, older adults have better self-control and less addictive behaviors [14,15]. So, could nonaddictive smartphone use help improve cognitive function in older adults and lower their risk of depression?

Furthermore, previous studies have identified abnormal brain activity in young individuals with smartphone addiction, such as a reduction in fronto-limbic resting-state functional connectivity and smaller gray matter volume in the right lateral orbitofrontal cortex [16,17]. According to it, we assume that there are also neural activity changes among older adults who use smartphones, which could be the potential mechanism explaining their cognitive performance.

Therefore, we designed this study to explore smartphone usage among older adults in a Chinese village and designed a cross-sectional study to explore: (1) the effect of smartphone use on cognitive function in older adults; (2) the effects of smartphone use on sleep and mental health; (3) the effect of smartphone use on brain activity among older adults.

## Methods

### Study Design and Participants

**Figure 1.**
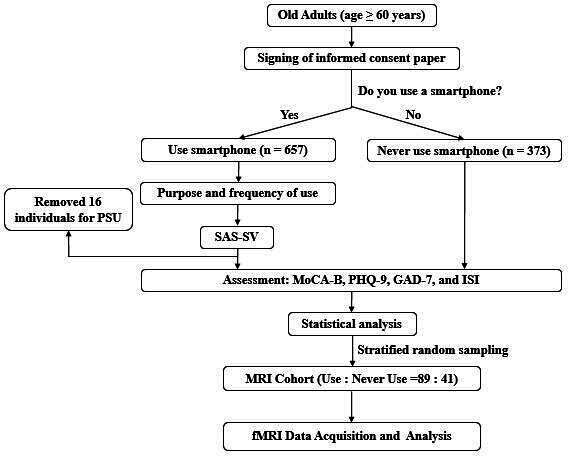
The flowchart of the whole study. fMRI: functional magnetic resonance imaging; GAD-7: Generalized Anxiety Disorder; ISI: Insomnia Severity Index; MoCA-B: Montreal Cognitive Assessment-Basic; MRI: magnetic resonance imaging; PHQ-9: Patient Health Questionnaire; PSU: problematic smartphone use; SAS-SV: Smartphone Addiction Scale-Short Version.

This study is a cross-sectional study, and the recruitment process was conducted as in the flowchart presented in Figure 1.

### Sample Size Calculation

For the appropriate sample size for the survey, we used G*Power (version 3.1; UC Regents), a widely recognized software tool for conducting power analyses. The calculation was based on a 2-tailed independent-samples *t* test, as we aimed to compare group differences in cognitive function (Montreal Cognitive Assessment-Basic [MoCA-B] scores) between smartphone users and nonusers. The effect size was estimated at Cohen *d*=0.3. The significance level (α) was set to .05, and the desired statistical power was .90, which is commonly used to ensure a sufficient likelihood of detecting true effects. A required sample size of 235 participants per group, with a total of 470 participants for both groups, was combined.

### Inclusion and Exclusion Criteria

This study was conducted from June 2023 to August 2023 in a village in Hubei, China, and all participants are registered local residents aged over 60 years. We recruited them by entrusting the village committee to spread an oral recruitment notice and then hired a fixed place in the village to complete the subsequent investigation. Data acquisition was completed by 7‐10 investigators with standardized training. A total of 1030 participants were initially screened for this study.

Inclusion criteria were the following: (1) voluntarily participating in this study and providing written informed consent; (2) participants were able to understand the meaning of the questions in the questionnaire and complete the questionnaire.

Exclusion criteria were the following: (1) a history of mental illness or a family history of mental illness; (2) recent major setbacks; (3) presence of endocrine or organic diseases; (4) Addictive mobile phone use (a total score of the Smartphone Addiction Scale-Short Version [SAS-SV] ≥31 for males or ≥33 for females).

### Ethical Considerations

The Ethics Committee of Renmin Hospital of Wuhan University approved this study. After a complete description of this study, written informed consent was obtained from all participants, and secondary analysis of the data was allowed. The research data are anonymous, and the privacy of the subjects will not be disclosed. Individual participants cannot be identified in any images of the manuscripts or supplementary materials. Each participant in this project received a compensation of CNY ¥50 (US $6.97) as a participation fee and CNY ¥20 (US $2.79) for travel reimbursement. The data for this study were collected prospectively to explore the effects of mobile phone use on older adults and were not part of any other study.

### Measures

First, general demographic data of all participants were acquired, including age, gender, and years of education. Subsequently, the symptoms of depression, anxiety, and insomnia were assessed by the Patient Health Questionnaire (PHQ-9) [18], Generalized Anxiety Disorder (GAD-7) [19], and Insomnia Severity Index (ISI) [20], respectively. The Cronbach α values for PHQ-9, GAD-7, and ISI were 0.89, 0.93, and 0.82, respectively, demonstrating good internal consistency [18,21,22].

For smartphone usage, participants were asked a question, “Do you use a smartphone in your daily life?” If the answer was no, the investigation of smartphone usage was concluded. If the answer was yes, participants were further queried about their most frequently used smartphone function and the average number of hours they spent using the smartphone per day. Additionally, the symptom of problematic smartphone use (PSU) was evaluated using SAS-SV [23]. A total score of SAS-SV ≥31 for males or ≥33 for females indicated PSU.

The cognitive function was assessed by the MoCA-B, which has demonstrated excellent validity in assessing cognitive function in poorly educated older adults [24]. The MoCA-B assessed cognitive domains of executive function, orientation, memory, abstraction, visual perception, and attention. The maximum score attainable is 30. To overcome the bias of education, 1 point was added for those participants with ≤4 years of education and 2 points for nonreaders (adjusted total scores ≤30).

### Statistical Analysis

A total of 16 participants were excluded due to PSU. Finally, a total of 1014 participants were included in the statistical analysis and subsequently divided into 2 groups, “use smartphone” and “never use smartphone.” All analyses were carried out in SPSS (version 26.0; IBM Corp).

First, the demographic characteristics were described using means and SDs or percentages. Differences between groups were assessed using independent sample *t* tests and chi-square tests. Then, the MoCA-B characteristics of the 2 groups were compared using Mann-Whitney *U* tests with Bonferroni correction. The severity of depression, anxiety, and insomnia was recorded ranging from 0 (none) to 3 (severe) or 0 (none) to 2 (severe), based on the scores of PHQ-9 (0‐3), GAD-7 (0‐2), and ISI (0‐3), respectively, and compared with Mann-Whitney *U* tests.

In our further investigation, participants were categorized based on their frequency of smartphone use. Considering that most older adults in this study use their smartphones within 3 hours per day, we divided them into 4 groups according to self-reported frequency of use: 1 hour per day, 2 hours per day, 3 hours per day, and more than 3 hours per day. Kruskal-Wallis tests were applied to compare those 4 groups in terms of the MoCA-B, PHQ-9, GAD-7, and ISI scores.

### Magnetic Resonance Imaging Data Acquisition

From the total sample of 1014 older adults who completed the questionnaire survey, a subsample of 130 participants was targeted for functional magnetic resonance imaging scanning. In addition to the exclusion criteria mentioned earlier, there were additional exclusion criteria for magnetic resonance imaging (MRI) analysis, such as the presence of metal objects in the body and claustrophobia. Among the eligible individuals, a stratified random sampling method was then used to select participants according to their smartphone usage status. If a selected participant declined MRI participation, another eligible individual was randomly selected to replace them. This procedure ultimately yielded a final sample of 130 participants, comprising 89 smartphone users and 41 nonsmartphone users.

Structural and functional MRI data were acquired on a 3-T United Imaging Healthcare scanner (uMR 780) at Three Gorges Uni RenHe Hospital. During the scanning, participants were instructed to remain still, stay awake, and avoid any structured cognitive activity. Resting-state functional magnetic resonance imaging data were acquired using a T2*-weighted echo-planar imaging sequence sensitive to blood-oxygen-level-dependent contrast [25,26]. Functional data were collected using the following parameters: repetition time=2000 ms, echo time=30 ms, flip angle=80°, matrix=64×64, and field of view=220 mm. A total of 240 volumes were obtained in 8 minutes and each volume consisted of 36 interleaved axial slices (slice thickness of 4 mm; no gap).

### MRI Data Analysis

MRI data analysis was performed using Statistical Parametric Mapping (version 12; Functional Imaging Laboratory) [27] and DPABI (Data Processing & Analysis for [Resting-State] Brain Imaging) [28] under MATLAB R2013b. Specifically, the data analysis workflow consisted of the following steps: (1) Quality control: to assess the data quality, we used Magnetic Resonance Imaging Quality Control tool [29], which examined various indicators such as signal-to-noise ratio, head motion, and ghost-to-signal ratio. (2) Preprocessing: the preprocessing steps included converting the data to Neuroimaging Informatics Technology Initiative format, removing the first 10 time points to ensure stability, conducting slice timing correction, realigning the images, segmenting structural images, normalizing the functional data, removing the linear trend of the time series, and regressing out nuisance variables. (3) Smoothing: we smoothed the images with a Gaussian kernel of 4 mm full width. (4) Calculation of amplitude of low-frequency fluctuations + fractional amplitude of low-frequency fluctuations. (5) Band pass filtering: a band-pass filter was used to extract signals in the conventional frequency band (0.01‐0.08 Hz). (6) Normalization: we normalized to a symmetric template and calculation of voxel-mirrored homotopic connectivity. (7) Calculation of regional homogeneity and degree centrality (with the correlation threshold set at *r*>0.25 [30]) using the unsmoothed data. (8) Two-sample *t* tests: these tests were performed to identify differences between two groups with the AlphaSim correction (voxel-wise threshold of *P*<.001 and cluster-wise threshold of *P*<.05). Age, gender, and education were included as covariates.

## Results

### Demographics and Smartphone Usage

As shown in Table 1, a total of 641 participants use smartphones in their daily lives, and 373 participants never use smartphones. There were no significant differences in age, gender, and the years of education between participants who used the smartphone and participants who never used the smartphone (*P*>.05). Within older adults who use the smartphone, the vast majority watch short videos on their smartphones (579/641, 90.3%), while the proportions for using social software, games and music, and other are 4.2% (27/641), 1.3% (8/641), and 0.6% (4/641), respectively.

**Table 1.** Demographic characteristics and smartphone usage of all participants.

	USm^a^ (n=641)	NUS^b^ (n=373)	2-tailed *t* test or chi-square^c^ (*df*)	*P* value
Age (years), mean (SD)	66.63 (4.18)	67.06 (4.51)	1.53 (1012)	.13
Sex, n			0.08 (1012)	.78
Male	264	157		
Female	377	216		
Education (years), mean (SD)	5.05 (3.64)	5.46 (3.69)	1.71 (1012)	.09
Frequency of use (hours/day), mean (SD)	1.81 (1.06)	N/A^d^	N/A	N/A
SAS-SV^e^, mean (SD)	19.63 (3.93)	N/A	N/A	N/A

a USm: use smartphone.

b NUS: never use a smartphone.

c A chi-square test was used for sex; a *t* test was used for all other variables.

d N/A: not applicable.

e SAS-SV: Smartphone Addiction Scale-Short Version.

### Depression, Anxiety, Insomnia, and Physical Pain

As shown in Table 2, there were no significant differences between the smartphone use group and the never-use-smartphone group in depression, anxiety, insomnia, and physical pain (*P*>.05).

**Table 2.** Depression, anxiety, insomnia, and physical pain of all participants. Analysis was completed using Mann-Whitney *U* tests.

	US^a^ (n=641), n (%)	NUS^b^ (n=373), n (%)	*z* score	*P* value
Depression level^c^			0.689	.49
None	567 (88.5)	324 (86.9)		
Mild	49 (7.6)	37 (9.9)		
Moderate	18 (2.8)	7 (1.9)		
Severe	7 (1.1)	5 (1.3)		
Anxiety level^d^			0.934	.35
None	604 (94.2)	347 (93)		
Mild	30 (4.7)	21 (5.5)		
Severe	7 (1.1)	6 (1.5)		
Insomnia level^e^			0.340	.73
None	480 (74.9)	284 (76.1)		
Mild	121 (18.9)	62 (16.6)		
Moderate	31 (4.8)	23 (6.2)		
Severe	9 (1.4)	4 (1.1)		

a USm: use smartphone.

b NUS: never use a smartphone.

c PHQ-9: Patient Health Questionnaire.

d GAD-7: Generalized Anxiety Disorder.

e ISI: Insomnia Severity Index.

### Cognitive Function

**Figure 2.**
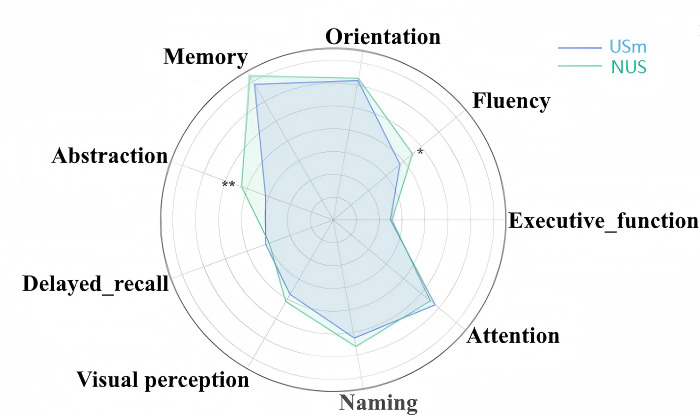
The difference in cognitive scores between the USm group and the NUS group. Scores for each domain were normalized before visualization to enable comparison across domains. USm: use smartphone; NUS: never use smartphone. * *P*<.005, ** *P*<.001.

In the smartphone use group, 325 (50.7%) participants added 1 point for ≤4 years of education, and 66 (10.3%) participants added 2 points for nonreaders. In the never-use-smartphone group, 128 (34.3%) participants added 1 point for ≤4 years of education, and 51 (13.7%) participants added 2 points for nonreaders. To overcome the bias of education, 1 point was added for those participants with ≤4 years of education and 2 points for nonreaders (adjusted total scores ≤30). Compared with those who never use the smartphone, people who use the smartphone have a better cognitive function assessed by MoCA-B, which was demonstrated in Table 3 and Figure 2. In addition to the total score (*z*=3.806, *P*<.001), people who use the smartphone have higher scores in subdomains of fluency (*z*=3.025, *P*=.002), naming (*z*=2.571, *P*=.01), abstraction (*z*=5.311, *P*<.001), and delayed recall (*z*=2.277, *P*=.02). Among those, the scores of total, fluency, and abstraction survived Bonferroni correction (*P*<.005). Depression, anxiety, insomnia, and cognitive characteristics of MRI participants are presented in Table S1 in Multimedia Appendix 1.

**Table 3.** Cognitive function of all participants.

Cognitive function	USm^a^ (n=641), mean (SD)	NUS^b^ (n=373), mean (SD)	*z* score	*P* value
Executive function	0.26 (0.44)	0.25 (0.43)	0.230	.82
Fluency	0.90 (0.73)	0.76 (0.72)	3.025	.002
Orientation	5.26 (0.44)	5.24 (0.44)	0.653	.51
Memory	2.46 (0.55)	2.37 (0.63)	1.767	.08
Naming	3.13 (0.44)	3.05 (0.46)	2.571	.01
Attention	1.66 (1.05)	1.74 (1.06)	1.180	.24
Abstraction	1.28 (0.97)	0.94 (0.82)	5.311	<.001
Delayed recall	2.19 (1.06)	2.25 (0.86)	2.277	.02
Visual perception	1.83 (0.64)	1.75 (0.69)	1.953	.051
Total score	19.66 (2.54)	18.98 (2.63)	3.806	<.001

a USm: use smartphone.

b NUS: never use a smartphone.

### Further Explore the Effects of Smartphone Use Frequency

As demonstrated in Table 4, there were no significant differences in MoCA-B and GAD-7 scores among the 3 groups with different frequencies of smartphone use. However, individuals who used their smartphones for ≥3 hours per day exhibit higher levels of depression (H=8.776, *P*=.03) and insomnia (H=13.335, *P*=.004).

**Table 4.** Clinical and cognitive characteristics of participants with different frequencies of smartphone use. Analysis was completed using Kruskal-Wallis tests.

		MoCA-B^a^	Means of ranks		
	Participants, n	Total score	PHQ-9^b^	GAD-7^c^	ISI^d^
Frequency (h/d)					
<1	315	19.60 (2.58)^e^	313.39	317.71	303.76
1‐2	165	19.79 (2.56)^e^	315.36	316.06	330.05
2‐3	134	19.63 (2.34)^e^	341.04	331.44	336.89
>3	27	19.89 (3.00)^e^	344.76	337.72	388.00
H-value	N/A^f^	0.348	8.776	5.284	13.335
*P* value	N/A	.95	.03	.15	.004

a MoCA-B: Montreal Cognitive Assessment-Basic.

b PHQ-9: Patient Health Questionnaire.

c GAD-7: Generalized Anxiety Disorder.

d ISI: Insomnia Severity Index.

e Mean (SD).

f N/A: not applicable.

### Brain Activity

**Figure 3.**
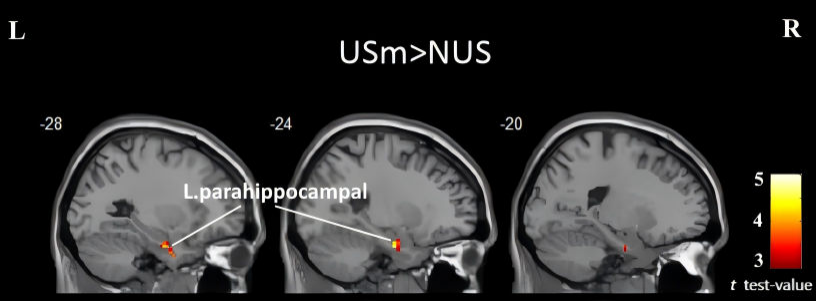
Brain regions showing significantly increased degree centrality in the USm group compared to the NUS group. The cluster in the left parahippocampal gyrus is visualized in sagittal slices at x=–28, –24, and –20 (MNI coordinates). The results were set at a threshold at voxel-wise *P*<.001 and cluster-level *P*<.05 (AlphaSim corrected). The color bar indicates *t* test values. L: left; MNI: Montreal Neurological Institute; NUS: never use smartphone; R: right; USm: use smartphone.

After excluding low-quality data, a total of 106 participants were included in the subsequent analysis, consisting of 75 smartphone users and 31 nonsmartphone users. No significant differences were observed between the 2 groups in terms of amplitude of low-frequency fluctuations, fractional amplitude of low-frequency fluctuations, regional homogeneity, and voxel-mirrored homotopic connectivity. However, in terms of degree centrality, older adults who were smartphone users exhibited higher values in the left parahippocampal gyrus (peak coordinates: x=−24, y=−9, z=−27; cluster size=20; *t*=4.52). The brain activity results have been presented in Figure 3. However, correlation analysis has not identified a correlation between the left parahippocampal gyrus’ degree centrality values and cognitive scores (Table S2 in Multimedia Appendix 1).

## Discussion

### Principal Findings

In this study, we have explored the effects of smartphone use on cognitive function and the relationship between smartphone use frequency and cognitive function and mental health among older adults. The main findings of this study were that nonaddictive smartphone use is associated with better cognitive function of older adults compared to those who never use smartphones and does not significantly affect sleep, depression, and anxiety levels but may increase the risk of depression and insomnia with further increases in smartphone use.

### Comparison to Prior Work

First, we have investigated smartphone usage among older adults. The results revealed that about 63.2% of older adults use smartphones in their lives, exceeding the internet penetration rate among older adults in 2021 (43.2%) [31] by nearly 20%, indicating the increasing prevalence of smartphones among older adults. Interestingly, we found that the most commonly used function among older adults was watching short videos (90.3%), which was inconsistent with the results of other studies [32,33]. Further inquiry revealed that most of them watched short videos on TikTok, which used to be regarded as the virtual playground of teenagers [34], indicating that older adults today are bridging the digital divide and exploring new technological platforms.

The prevalence of PSU in this study was relatively low (approximately 2.4%), consistent with the findings of Busch et al [35]. This could be attributed to the fact that, compared to younger individuals, older adults tend to concentrate their smartphone usage on a limited range of recreational activities (such as TikTok rather than gaming or others), and their work does not typically require smartphone involvement. Furthermore, during this study, we also found that older adults may experience difficulties maintaining prolonged smartphone usage due to physical discomfort. They reported experiencing pain in the neck and wrist, which prevented them from using smartphones continuously, even if they desired to continue it. This means that physical pain may be an important reason for the low rate of mobile phone addiction in older adults, but at the same time, it suggests that smartphone use may have adverse physical effects on older adults, which needs to be further explored in future investigations.

As regards nonaddictive smartphone use, our study demonstrated that older adults who incorporate smartphones into their daily lives exhibited better cognitive function than those who never use smartphones. Several other similar studies have also reported similar findings, highlighting the positive effects of smartphone use on cognition in older adults [32,36,37]. Using smartphones could be regarded as a mentally stimulating activity. According to the “use it or lose it” hypothesis, more involvement in mentally stimulating activities would bring less age-related cognitive decline [38], and the involved mechanisms may include increasing functional connectivity within related brain networks [39] and promoting neural plasticity [40,41]. Specifically, in our study, older adults who used smartphones displayed better performance in fluency and abstraction. Increased social engagement and reduced feelings of loneliness [42] could improve the verbal fluency of smartphone users. Moreover, older adults venturing into more advanced smartphone features, such as short videos, may experience heightened cognitive load and increased brain activation [43], potentially facilitating the enhancement of advanced cognitive functions, including abstraction.

Among all resting-state MRI indicators, our study discovered that only, in terms of degree centrality, older adults who were smartphone users exhibited higher values in the left parahippocampal gyrus. Degree centrality is an analytical method that evaluates the local properties of resting-state functional magnetic resonance imaging signals by measuring the extent to which a given brain voxel (node) is directly connected to other voxels [44,45]. The parahippocampal gyrus is known to be involved in various cognitive processes, including scene recognition, episodic memory, spatial navigation, and memory encoding and retrieval [46]. This finding aligns with another outcome obtained in this study, which demonstrates that older adults who use smartphones exhibit better performance in delayed recall. However, this effect was nonsignificant after correction for multiple comparisons. As memory is a crucial component of cognitive function and tends to decline with age, one possible explanation is that smartphone use may stimulate the parahippocampal gyrus and potentially enhance memory in older adults. Furthermore, regarding certain specific brain areas such as the middle frontal gyrus, as well as the lingual gyrus, which were previously found to be possibly associated with smartphone use in prior studies [47,48], this current investigation did not demonstrate clear activation or deactivation. Overall, the neural activation patterns observed in individuals using smartphones in this study were far less significant compared to previous findings. One possible explanation for this discrepancy is the emphasis on nonaddictive usage and a relatively shorter duration of smartphone usage, as repeatedly underscored throughout our research.

This study also found that healthy smartphone usage does not have a significant impact on the sleep quality, depression, and anxiety levels of older adults. As previously mentioned, the detrimental effects of smartphones on sleep and psychological well-being largely stem from unhealthy patterns of use. For example, using smartphones for long periods or at night [49,50] can considerably impair their sleep and increase the risk of depression. However, for older people, possibly because of physical limitations (excessive smartphone use can cause physical discomfort) [51], they rarely show excessive or unhealthy smartphone use and often get satisfaction from it [9,52]. These factors may be important reasons to protect them from the adverse risks of excessive smartphone use.

It is worth mentioning that as cell phone use further increased, it did not seem to continue to improve cognitive performance in older adults, but instead led to higher levels of insomnia and depression. This is consistent with previous research on young adults [49,53], suggesting that long-term use of mobile phones can also adversely affect mental health and sleep quality in older adults.

### Limitations

A few limitations of this study also need to be taken into consideration. First, this study is a cross-sectional study and could not prove the causal relationship between smartphone use and cognitive function among older adults. So we plan to conduct follow-up assessments on the participants in this study in the future. Second, although we aimed to investigate all eligible older adults in the village to conduct a census, a small proportion of older adults were unable to participate due to factors such as physical limitations and personal reasons. This may introduce bias to the results to a certain extent. Third, all of the cognitive domains explored in this study fall within the realm of neurocognition, and another important aspect of cognition, namely social cognition [54], has not been assessed. Additionally, we determined the minimum required sample size for this survey through sample size calculation; however, for the MRI component, the sample size was determined based on prior experience, which may result in insufficient statistical power.

### Future Directions

Our study could provide a reference for further studies to investigate the underlying mechanisms through which smartphones improve the cognition of older adults and develop interventions that leverage smartphone use to improve cognitive function. Considering that cognition is strongly associated with the function of brain regions [55], future studies could integrate other modal imaging measures, such as task-related functional magnetic resonance imaging, to further explore the potential mechanisms underlying the cognitive benefits observed in older adults who use smartphones.

### Conclusions

In sum, this study reveals that older adults who incorporate smartphones into their daily lives demonstrate better cognitive function compared to those who never use smartphones. However, excessive smartphone use is associated with an increased risk of mental health problems instead of improved cognitive function. Based on our findings, we suggest that short periods of daily smartphone use can improve cognitive function in older adults without damaging mental health.

## Supplementary material

10.2196/63485 Multimedia Appendix 1 Information of MRI participants. MRI: magnetic resonance imaging.

## Abbreviations

DPABI: Data Processing & Analysis for (Resting-State) Brain Imaging
GAD-7: Generalized Anxiety Disorder Scale
ISI: Insomnia Severity Index
MoCA-B: Montreal Cognitive Assessment-Basic
MRI: magnetic resonance imaging
PHQ-9: Patient Health Questionnaire
PSU: problematic smartphone use
SAS-SV: Smartphone Addiction Scale-Short Version
