# Supplementary material for: Cognitive and Spontaneous Brain Activity in Nonaddictive Smartphone Users Among Older Adults in China: Cross-Sectional Study
Source: J Med Internet Res. 2025 Jul 24;27:e63485. doi: 10.2196/63485 (PMC12288861; doi:10.2196/63485)
Supplement: Multimedia Appendix 1 — Information of MRI participants. MRI: magnetic resonance imaging. [file jmir-v27-e63485-s001.docx]

**Table S1**. Depression, anxiety, insomnia and cognitive characteristics of MRI participants.

|  | **US (N = 75)** | **NUS (N = 31)** | **Z** | ***P* Value** |
| --- | --- | --- | --- | --- |
| **Depression level** | 1.99 ± 3.387 | 2.48 ± 3.723 | 0.854 | .39 |
| **Anxiety level** | 0.67 ± 1.905 | 0.97 ± 2.677 | 0.301 | .76 |
| **Insomnia level** | 4.99 ± 5.374 | 5.48 ± 6.371 | 0.141 | .89 |
| **Cognitive function** |  |  |  |  |
| Executive function | 0.31 ± 0.464 | 0.39 ± 0.495 | 0.797 | .43 |
| Fluency | 1.03 ± 0.788 | 0.81 ± 0.703 | 1.327 | .19 |
| Orientation | 5.43 ± 0.498 | 5.26 ± 0.445 | 1.621 | .11 |
| Memory | 2.56 ± 0.500 | 2.42 ± 0.502 | 1.312 | .19 |
| Naming | 3.07 ± 0.553 | 2.68 ± 0.653 | 3.039 | .002 |
| Attention | 1.77 ± 1.034 | 1.74 ± 1.032 | 0.126 | .90 |
| Abstraction | 1.76 ± 0.928 | 1.65 ± 1.082 | 0.429 | .67 |
| Delayed recall | 2.63 ± 0.941 | 2.13 ± 0.846 | 2.314 | .02 |
| Visual perception | 2.15 ± 0.748 | 1.68 ± 0.599 | 2.988 | .003 |
| Total score | 20.69 ± 2.033 | 18.74 ± 1.612 | 4.342 | <.001 |

Data are shown as mean ± S.D. US, use smartphone; NUS, never use smartphone.

**Table S2**. Relationship between the left parahippocampal gyrus' degree centrality values and cognitive scores among older adults who use smartphones.

|  | **r** | ***P* Value** |
| --- | --- | --- |
| Executive function | 0.111 | .35 |
| Fluency | 0.121 | .31 |
| Orientation | 0.070 | .56 |
| Memory | 0.066 | .58 |
| Naming | -0.086 | .47 |
| Attention | 0.027 | .82 |
| Abstraction | -0.121 | .31 |
| Delayed recall | -0.017 | .89 |
| Visual perception | 0.095 | .43 |
| Total score | 0.069 | .57 |
